# Antibody- Based Immunotherapy Combined With Antimycotic Drug TMP- SMX to Treat Infection With *Paracoccidioides brasiliensis*

**DOI:** 10.3389/fimmu.2021.725882

**Published:** 2021-10-19

**Authors:** Camila Boniche-Alfaro, Brenda Kischkel, Luciana Thomaz, Monique Maria Carvalho-Gomes, Leila M. Lopes-Bezerra, Joshua Daniel Nosanchuk, Carlos Pelleschi Taborda

**Affiliations:** ^1^ Instituto de Ciências Biomédicas, Departamento de Microbiologia, Universidade de São Paulo, São Paulo, Brazil; ^2^ BIDiagnostics, Centro de Inovação, Empreendedorismo e Tecnologia (CIETEC)/Universidade de São Paulo, São Paulo, Brazil; ^3^ Department of Medicine (Division of Infectious Diseases), Microbiology and Immunology, Albert Einstein College of Medicine, New York City, NY, United States; ^4^ Laboratory of Medical Mycology, Institute of Tropical Medicine of São Paulo, Department of Dermatology, School of Medicine, University of São Paulo, São Paulo, Brazil

**Keywords:** antibody therapy, monoclonal antibodies, immunotherapy, fungal cell wall glycoconjugates, infectious diseases, systemic mycosis, paracoccidioidomycosis

## Abstract

Monoclonal antibodies (mAbs) are promising alternatives to treat infectious diseases, especially given their potential for applications in combination therapies with antimicrobial drugs to enhance the antifungal efficacy. Protection mediated by mAbs used to treat experimental paracoccidioidomycosis (PCM) has been demonstrated previously. Our aim in the present work was to characterize a monoclonal antibody (mAbF1.4) raised against a cell wall glycoconjugate fraction of *Paracoccidioides* spp. and to analyze its efficacy combined with trimethoprim-sulfamethoxazole (TMP/SMX) as treatment for experimental PCM. We demonstrated that the epitope recognized by mAbF1.4 is consistent with branched glucose residues present on a cell wall β-glucan polymer. *In vitro*, mAbF1.4 increased the phagocytic capacity and nitric oxide concentration induced by the macrophage cell line J774.1A, and this resulted in a significant reduction in the viability of the opsonophagocytized yeasts. *In vivo*, we detected a significant reduction in pulmonary fungal burdens of mice treated with mAbF1.4 in association with TMP/SMX, which correlated with increased pulmonary concentrations (determined by ELISA) of IFN- γ, TNF-α, IL-10 and IL-17. In parallel, we observed a decrease in IL-4, suggesting that the treatment was associated with a mixed Th1-Th17 type immune response. Histopathology of lung segments from mice receiving the combination therapy showed a significant reduction in granulomas, which were well-defined, and improved maintenance of lung architecture. These findings demonstrate that mAbF1.4 + TMP/SMX therapy is a promising approach to combat PCM as well as decrease disease sequelae and highlights the potential benefits of immune mediators in PCM combined immunotherapy.

## Introduction

Monoclonal antibodies (mAbs) are immunotherapeutics commonly used in the treatment of cancer and immune system disorders. They are also promising alternatives to treat infectious diseases, and in addition to their direct action on microbes, mAbs might potentially enhance the efficacy of other antimicrobial drugs to eliminate multidrug-resistant microorganisms. The administration of mAbs can mitigate host damage, reduce the duration of antimicrobial therapies and lower their toxicity effects, increasing compliance with the drug regimen (1–3). In certain patients, conditions such as natural or acquired immunosuppression, and lack of antibody production may interfere with the efficiency of chemotherapies (4, 5), making those individuals key candidates for mAb immunotherapy in combination with antimicrobial agents. Currently, the majority of the clinically applied mAb therapies are of human IgG1 subtype (6, 7). However, there is no approved mAb based immunotherapy to treat fungal infections in humans or animals (1; van 2, 5).

Some infectious diseases require long-term antimicrobial drugs to achieve complete recovery. Such is the case of chronic paracoccidioidomycosis (PCM), a systemic mycosis characterized by a pulmonary granulomatous reaction. PCM is caused by thermal dimorphic fungi of the *Paracoccidioides* genera, and it is prevalent in rural areas in Latin America, from Mexico to Argentina, but especially in Brazil, where the highest prevalence and morbidity rates are registered (8, 9). Chronic PCM treatment is based on the administration of antifungal drugs such as sulfonamides, azole derivatives and amphotericin B, for extended periods of time (minimum ~1.5 years), depending on the severity of each patient’s clinical condition. A lack of adherence to the PCM treatment is the most common reason of therapeutic failure, primarily due to patient fatigue with the constant use of antifungal drugs for extended periods (8, 10). Furthermore, renal toxicity caused by antimycotic drugs, resistance of some *Paracoccidioides* spp. isolates to the available antifungal agents, and the relatively high chances of relapse underscore the urgent need to study and develop alternative therapeutic options to treat PCM (11, 12). Several studies have demonstrated protection mediated by mAbs. Immunotherapy to treat experimental PCM has been performed using different subtypes of murine mAbs to glycoproteins and heat shock proteins, resulting in protective responses, which are characterized by reduced pulmonary CFU, decreased lung damage, and enhanced production of pulmonary cytokines, associated with a Th1 type immune response (13–16). Therefore, we have proposed that mAb immunotherapeutics to treat PCM may enhance the protective effect of antimycotic drugs, reduce the fibrotic consequences of disease, shorten the required time of treatment, and, possibly, prevent recurrences.

Fungal antigens present in the cell wall are desirable targets for drug design and development, since they are accessible to the immune system and vital for many processes such as growth, virulence and pathogenicity (17). Previously described protective antibodies to fungal pathogens mostly recognized surface antigens (18). β-glucans have been proposed over 40 years as desirable targets for antifungal immunotherapy, since these components are not present in mammalians cells (1), and because polysaccharides from the fungal cell wall interact with immune receptors and play key roles in the host immune responses (19). In this work, we generated and characterized a new mAb to a *Paracoccidioides brasiliensis* cell wall glycoconjugate and determined the ability of that mAb to modify experimental PCM. This is the first report of a combined therapy based on protective monoclonal antibodies to a *P. brasiliensis* cell wall glycoconjugate and an antimycotic drug modulating experimental PCM.

## Materials and Methods

### Ethics Statement

All procedures were performed according to the guidelines of National Council of Ethics with Animals (CONCEA) and the animal study protocols were approved according to Animal Use Ethics Committee (CEUA- ICB protocol number 66/2017) at Universidade de São Paulo.

### Cell Wall Glycoconjugate Extraction and mAbF1.4 Generation

We extracted a soluble cell wall glycoconjugate fraction of *P. brasiliensis* yeasts using alkaline hydrolysis with 2% KOH (20). The soluble extract was analyzed by gas chromatography (**Supplementary Table 1**), which showed that it was largely comprised of polysaccharides (11% mannose, 7% galactose and 79% glucose). Then, 6-week-old female BALB/c mice were immunized 8 times intraperitoneal route with 100 μg of glycoconjugate fraction using 10 µg of Quil A (*Quillaja saponaria* Molina cortex) as adjuvant (immunizations were repeated every 7 days for 8 weeks. A boost immunization was applied two days before the spleen harvest at week 8). The antibody titers were screened by ELISA. Several IgM and an IgG mAb cell lines (mAbF1.4) were obtained by hybridoma technology at the Hybridoma Facility Center of the Albert Einstein College of Medicine according to published protocols (21). The reactivity of the mAbs obtained against the glycoconjugate fraction was confirmed by ELISA (**Supplementary Figure 2**) and mAbF1.4 was selected for further studies because of its highest binding efficiency.

### MAbF1.4 Isotype Identification

The isotype of mAbF1.4 was identified by a commercial ELISA (mouse immunoglobulin isotyping ELISA kit BD Pharmigen™) according to the manufacturer protocol. Different samples of mAbF1.4 purified solutions were tested in triplicate for murine immunoglobulin IgG isotypes 1, 2a, 2b and 3. Plates were read on Epoch 2 BioTek^®^ spectrophotometer at 450 nm.

### Yeasts and Grow Conditions

The virulent *P. brasiliensis* (Pb18) yeast isolate was obtained from Instituto de Medicina Tropical da Faculdade de Medicina da Universidade de São Paulo. To keep the yeasts growing for the inoculum preparation, the isolates were transferred from Fava Neto agar to Fava Neto broth supplemented with 10% heat inactivated FBS (LGC Biotecnologica) plus 0.1% of Gentamicin (LGC Biotecnologica) and incubated at 37°C under constant agitation at 150 rpm for 6-8 days. For the inoculum preparation, yeast cultures were washed three times with sterile PBS. Viability of the fungal cells was determined by counting live/dead cells in a hemocytometer with 0.4% Trypan Blue (Sigma-Aldrich) exclusion staining. The concentration of the inoculum was adjusted to 3,0 ×10^5^ yeast/50 μL for intratracheal infection or 1,5 x10^5^ per well for phagocytosis assays.

### Antibody Purification

In addition to the mAb F1.4 hybridoma, we used the mAb 4G2 cell line (ThermoFisher Scientific) to generate the IgG control mAbs used in all the experiments. Antibodies were produced weekly over 6 months from the respective hybridomas grown in culture flasks (Kasvi) fed with low- glucose Dulbecco′s Modified Eagle′s Medium (DMEM), plus 2% non-essential amino acids, 2% HEPES, 2% NaHCO3, 2,5% sodium pyruvate and, 0,1% gentamicin sulphate (LGC Biotecnologica) and supplemented with 20% FBS. Antibodies were purified using Pierce™ Recombinant Protein A/G immunoaffinity chromatography (Thermo Fisher Scientific) per the manufacturer’s instructions. Eluted antibodies were neutralized in Tris-HCl to final concentrations of 100 mM, concentrated by centrifugal filtration (30K Amicon), filter sterilized, and stored at ^-^20°C until use. Concentrations were determined by absorbance at 280 nm, which correlated with Bradford assay results.

### Dot Blot for mAbF1.4 Epitope Screening

For dot blotting, samples (100 μL) of laminarin (Sigma-Aldrich), zymosan (Sigma-Aldrich), yeast mannan (Sigma-Aldrich), soluble β-glucan extract from *Saccharomyces cerevisiae* obtained using published protocols (22, 23) and Pb18 cell wall glycoconjugate extract (as a positive control) at a concentration of 0.1 μg/μL (w/v) were spotted onto nitrocellulose membrane, which were covered with aluminum foil and allowed to dry overnight at 4°C, according to Vink et al. with minor modifications (24). Primary antibodies (mAbF1.4 and IgG control) were diluted in 1% PBS-BSA buffer to a concentration of 0.1 μg/μL, and 100 μL of this buffer were applied to each well. Then, the membrane was incubated at 37°C for 2 hours. Three washes were performed with TBS (PBS- 0.05% Tween 20) and the membranes were dried by vacuum. Next, the membranes were blocked for 90 minutes with 3% PBS-BSA. Then, the membranes were probed with ECL Prime Anti-mouse IgG Horseradish Peroxidase (GE Healthcare) at a dilution of 1:2500 in 1% PBS-BSA buffer. The reactions were visualized with ECL™ Prime Luminol Enhancer and Peroxide Solution (GE Healthcare) and immediately imaged in a documentation system. The experiment was performed in triplicate and repeated three independent times. For the binding quantification analysis, we performed the gel analysis tool on ImageJ, for determination of area. Data were presented as mean ± SEM and analyzed with the two-tailed Student’s t-test between each polysaccharide and the Mannan values, since mAbF1.4 does not bind Mannan.

### Macrophage Phagocytosis

Macrophage cell line J774.1A (ATCC) was used for phagocytosis assays according to Buissa Filho *et al*. with minor modifications (15). For each experiment, 1.5 x10^5^ cells per well were plated into 24-well culture plates (TPP). Macrophages were activated with recombinant murine IFN-γ (50 U/ml, BD Biosciences) and incubated at 37°C in 0,05% CO_2_ atmosphere, overnight. The medium in each well was then replaced by the following additions in a proportion of 5 macrophages to 1 yeast cell; (1) yeasts cells of *P. brasiliensis* (Pb18**)** opsonized for 2 hours with mAbF1.4 (10µg); (2) yeasts cells of Pb18 opsonized for 2 hours with the irrelevant mAb (10µg); (3) Pb18 yeasts cells. This step was followed by an incubation at 37°C for 12 hours. The supernatants were collected for NO determination and every well was washed three times with PBS, followed by three washes with 0.15 M α-mannopyranoside (Sigma Aldrich) to remove non-internalized yeasts. The cells were fixed for 30 minutes with cold absolute methanol, and finally then stained with Giemsa (Laborclin) to calculate the *Phagocytic index* (PI), which is defined as


PI=P∗F

Where P is the percentage of macrophages with internalized yeasts and F the average of phagocyted yeast cells, counted by light microscopy at 400X magnification. Experiments were performed in triplicate and different fields were counted until 1000 cells were checked. Finally, in order to visualize the phagocytosis assay, we used immunofluorescence staining.

### Macrophages Antifungal Activity

After incubation within the macrophages, the viability of the opsonophagocyted yeasts was determined by plating the content of the macrophages after hypotonic lysis with sterile distillated H_2_O on BHI agar supplemented with 4% FBS and 5% culture filtrate of 192 strain *P. brasiliensis*, and 0.1% of gentamicin sulphate. CFU were counted after 21 days of incubation at 37°C. Experiments were performed in triplicate.

### Nitric Oxide Production by Macrophages

The levels of Nitric Oxide (NO) were determined by Griess reaction, assessed as the nitrite present in the culture supernatant of challenged macrophages, according to published protocols (25). In 96 well plates, 100 μL of every sample plus 50 μL of Griess reagent (0,1% N-1 naftil-etil-enediamine, 1% sulfanilamide, 2,5% phosphoric acid) were applied. Calibration curves were performed using NaNO_2_ standard solutions (100- 1,5625 µM). Absorbance was determined at 550 nm. Experiments were performed in triplicate.

### Immunofluorescence Staining to Verify Phagocytosis

To visualize the phagocytosis assay, we performed an immunofluorescence staining. Before the 12-hour phagocytosis incubation*, P. brasiliensis* yeasts were stained with Calcofluor White (10 μg/ml; Sigma-Aldrich) at room temperature, for 15 minutes, under agitation and covered with aluminum foil. Next, a secondary antibody (anti-mouse IgG labeled with Alexa Fluor 488 nm; Life Technologies) diluted 1:2500 was added to the yeasts (1.5 x10^5^ cells per well) for two hours at 37°C. The yeasts were co-incubated with macrophages, as above, for 11 hours at 37°C, in 0,05% CO_2_ atmosphere. Finally, the plate was incubated with a lysosome labeling compound according with Lysosomal Staining Kit Cytopainter (Abcam) for 1 hour to evaluate the formation of phagolysosomes within live macrophage cells. Our goal was to visualize by immunolabeling the phagocytosis of the opsonized and non-opsonized yeasts. Calcofluor white staining was detected on DAPI channel. The formation of antigen- antibody complexes was observed on GFP channel. Cytopainter labeling was visualized on RFP channel. The images were obtained on a EVOS fluorescence microscope (ThermoFisher Scientific) at 100X.

### Animals

Male C57Bl/6 mice (6 to 8-week-old) were bred at the Faculdade de Medicina da Universidade de São Paulo (FMUSP) animal facility, under specific pathogen-free conditions. The mice were kept in the Department of Microbiology, Instituto de Ciências Biomédicas da Universidade de São Paulo (ICB USP) animal facility and had access to water and food *ad libitum*. In order to cause chronic infection, C57Bl/6 mice were inoculated intratracheally with virulent *P. brasiliensis* (Pb18) yeast cells according to Taborda, et al. (26). Briefly, animals were anesthetized intraperitoneally with 300μl of a solution containing 80 mg/kg ketamine (Vetnil) and 10 mg/kg of xylazine (Ceva), and, after verifying that the mice showed no reaction to test stimuli, the mouse’s neck was hyperextended, and the trachea exposed to inoculate 50μL of PBS containing 3,0 ×10^5^ yeasts cells using a 26-gauge needle. The incisions were sutured with 5-0 silk and the mice were kept warm until recovery.

### Combined Immunotherapy

Groups (n = 5) were randomly organized: (i) infection control: mice infected with 3,0 ×10^5^ *P. brasiliensis* (Pb18) yeast cells; (ii) Pb 18 infected and treated with TMP/SMX; (iii) Pb 18 infected and treated with mAbF1.4, (iv) Pb 18 infected and treated with TMP/SMX + mAbF1.4 (400 µg); (v) infected and treated with TMP/SMX + irrelevant mAb (mAb4G2; 400 µg); (vi) Sham (uninfected, untreated). For the TMP/SMX groups, at 30 days of infection, the animals were treated intraperitoneal with TMP/SMX (NeoQuímica) at 15 mg/kg/SMX: 3 mg/kg dose daily. For mAb treatment groups, at 30 days post-infection, mAbF1.4 or mAb4G2 (100 µg) was administered weekly for 4 weeks. At day 58, the animals were euthanized, and the lungs were sterilely extracted and processed. The experiment was performed in triplicate.

### Evaluation by Fungal Burden

Treatment efficacy was evaluated by counting colony forming units (CFU) from viable yeasts present in lungs recovered at 58 days of infection. For this experiment, after euthanasia, lungs were aseptically removed. Transversal sections of the tissue were randomly collected for histological processing, and the remaining tissue was weighted to calculate CFU/gram. The lungs were then homogenized in 2 mL of PBS and 100 μL were plated into BHI agar supplemented as described above (macrophages antifungal activity). CFUs were counted manually at 21 days of incubation at 37°C.

### Evaluation of the Pulmonary Cytokine Profile

For cytokine quantification, 1000 μL of the lung homogenates were aliquoted into microtubes containing 1000 μL of protease inhibitor (Protease Inhibitor Panel (INHIB1) Sigma-Aldrich) using the following recipe: Pepstatin A (P5318)—50 µg/mL, Benzamidine HCl (B6506)—50 mg/mL, N-Ethylmaleimide (E3876)—15.5 mg, EDTA (ED2SS)—1 ml−100 mM and distilled water for 50 ml of protease inhibitor solution. The samples were then centrifuged, and the supernatants frozen at -80°C until tested. The supernatants were assayed for cytokines IL-4, IL-10, IL-17, TNF-α and IFN-γ by Enzyme-Linked Immunosorbent Assay (ELISA) using commercial kits (OptEIA™, BD) according to the manufacturer’s protocols.

### Evaluation by Lung Histopathology

Sections of lung tissue were fixed in 10% buffered formalin and embedded in paraffin for sectioning. The sections were attached to slides and stained with hematoxylin and eosin staining (HE) to evaluate the morphology and the granuloma formation. The slides were also stained by Gomori- Grocott methenamine silver staining to better visualize the fungal cells (data not shown).

### Statistical Analysis

GraphPad Prism 9.1 software was used for statistical analysis of the phagocytosis assays, fungal burdens, and cytokine production. Datasets were compared by one-way ANOVA and a Tukey’s post-test.

## Results

### MAbF1.4 Isotype Identification

We used an ELISA assay to isotype the purified mAbF1.4. The mAbF1.4 was classified as IgG1 subtype (**Table 1**).

**Table 1:** Isotyping Elisa of purified mAbF1.4 solutions.

IgG Isotype	Absorbance (450 nm)		
	mAbF1.4	Kit Positive Control	Kit Negative Control
**IgG1**	**0,721**	0,633	0,056
IgG2a	0,061	0,427	0,059
IgG2b	0,033	0,279	0,042
IgG3	0,077	0,857	0,085
Blank	0,059	0,065	0,041

Different samples of purified mAbF1.4 solutions were tested in triplicate for defining the murine immunoglobulin IgG isotype as either 1, 2a, 2b or 3. Mouse immunoglobulin isotyping ELISA (BD Pharmigen™) plates were read on an Epoch 2 BioTek^®^ spectrophotometer at 450 nm. Grey highlight indicates the positive IgG1 reaction.

### Dot Blot for mAbF1.4 Epitope Screening

**Figure 1:**
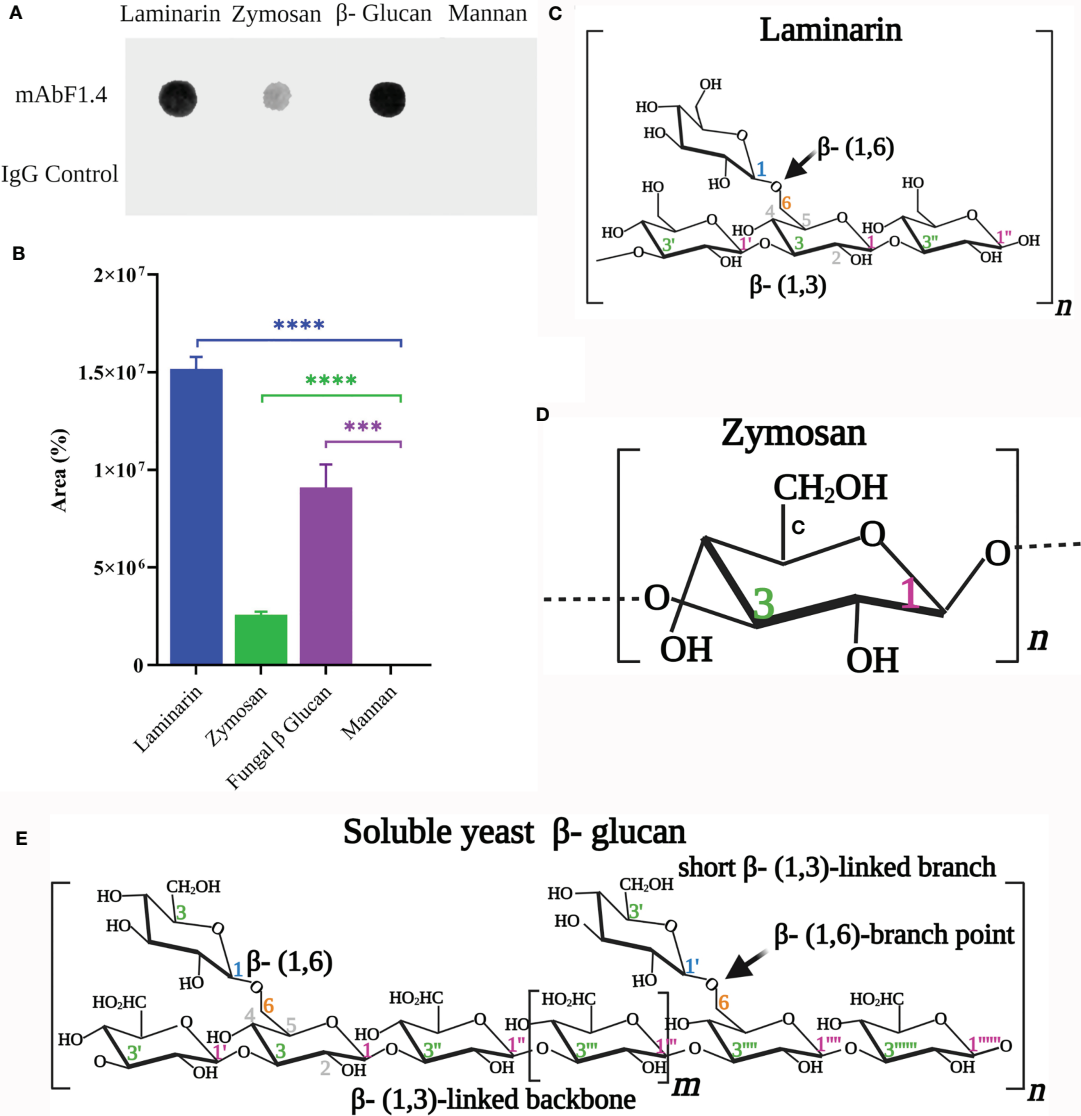
Partial identification of the mAbF1.4 epitope. **(A)** Dot blotting: samples (100 μL) of laminarin (0.1 μg/μL), zymosan (0.1 μg/μL), yeast mannan (0.1 μg/μL) and soluble β-glucan extract from *Saccharomyces cerevisiae* were tested with mAbF1.4 (10 μg). An IgG control (10 μg) was used as a negative control. An anti-mouse IgG Horseradish Peroxidase at 1:2500 dilution was visualized with Peroxide and immediately imaged in a documentation system. The dark spots in Laminarin, Zymosan and soluble β-glucan extract reactions with mAbF1.4 evidence antibody- antigen complexes. **(B)** Dot Blot Quantification analysis using the gel analysis tool on ImageJ for determination of area. Data were presented as mean ± SEM and analyzed with the two-tailed Student’s t-test between each polysaccharide and the Mannan values, since mAbF1.4 does not bind Mannan. Representation of the structures of **(C)** Laminarin, **(D)** Zymosan and **(E)** Soluble yeast β-glucan, all modified from Waszkiewics- Robak (27) and created with Biorender.com. ***p: 0.0003; ****p < 0.0001.

In **Figures 1A, B**, we identified that mAbF1.4 strongly reacted with laminarin (**Figure 1C**: β-1,6-, β-1,3-linked glucose polymer). A weak reaction was observed with zymosan (**Figure 1D**), which is a linear polymer of β-1,3-linked glucose polymer. Our results strongly suggest that mAbF1.4 recognizes the β-1,3-/β-1,6-linked glucose branches present in S. cerevisiae β-glucan extract (**Figure 1E**), which are absent in zymosan. Additionally, we identified that mAbF1.4 was unable to bind S. cerevisiae mannan. Our IgG control showed no reaction with neither of the polysaccharides, as shown in **Figure 1A**. In **Figure 1B** we performed quantification of the dot blot results using the gel analysis tool on ImageJ. The binding percentages were normalized using mannan-binding values, since mAbF1.4 does not bind to mannan. mAbF1.4 showed higher binding with Laminarin and Fungal β-glucan (S. cerevisiae β-glucan extract) (1,5x107% and 9,5x106 %, respectively) than with zymosan (2,6x105%). The experiment was performed in duplicate and repeated three independent times.

### Phagocytosis Assay, Macrophages Antifungal Activity, and Nitric Oxide Production

**Figure 2:**
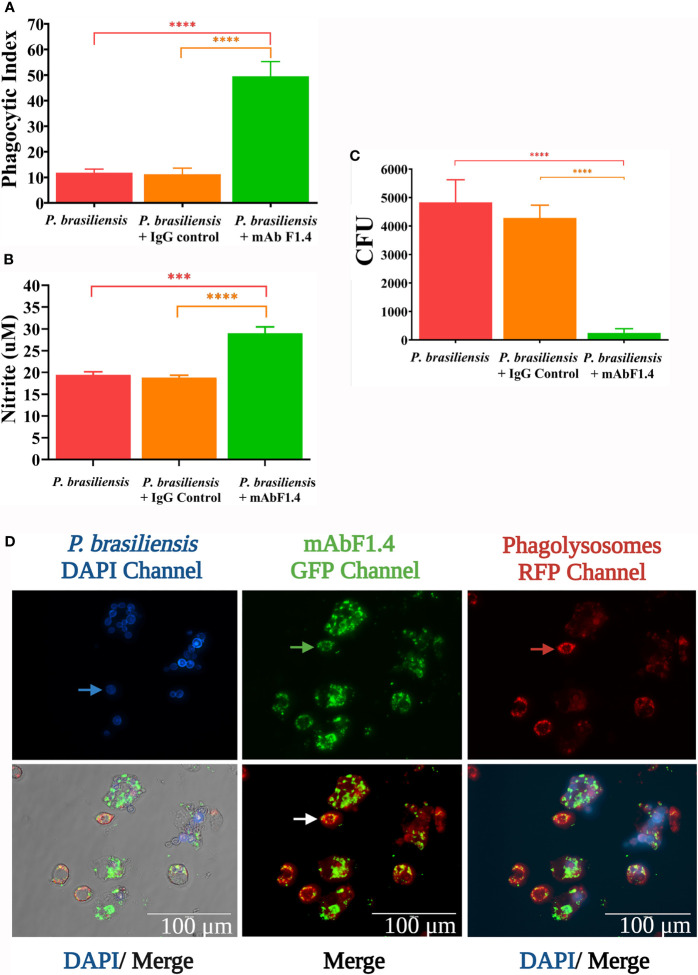
Phagocytosis assays performed with J774.1A cell line macrophages challenged with *P. brasiliensis* (Pb18) with or without opsonization with mAbF1.4 or the IgG control mAb. Bars depict means and SEMs of three independent experiments, with wells performed in triplicate. Overall differences in the variance of all treatments were assessed for significance by repeated-measures one-way ANOVA with results of Tukey’s *post hoc* test for multiple comparisons displayed in the graph. ****P < 0.0001. **(A)** Phagocytic index assessed as PI=P*F. Where P is the percentage of macrophages with internalized yeasts and F the average of phagocyted yeast cells, counted by light microscopy at 400X magnification. Experiments were performed in triplicate and different fields were counted until 1000 cells were checked. **(B)** NO determination as by Griess reaction, assessed as the nitrite present in the culture supernatant of challenged macrophages. Calibration curves were performed using NaNO_2_ standard solutions (1,5625 - 100 µM). Absorbance was determined at 550 nm. **(C)** Killing effect determined by the viability of the phagocyted yeasts by plating the content of the macrophages after hypotonic lysis with sterile distillated H_2_O on BHI supplemented agar. CFU were counted after 21 days of incubation at 37°C. **(D)** Phagocytosis assay visualized by immunofluorescence. *P. brasiliensis* yeasts were stained with Calcofluor White, visible in the DAPI channel. The blue arrow points out non- opsonized/non- internalized yeasts. The formation of antigen- antibody complexes were revealed with an anti-mouse IgG labeled with Alexa Fluor 488 nm and observed in the GFP channel. The green arrow points out opsonized/internalized yeasts. Lysosome labeling was used to evaluate formation of phagolysosomes within live macrophage cells and were visualized in the RFP channel. The red arrow shows macrophage lysosomes. In the merge images the channels were overlayed to integrate the information. The photomicrographs were obtained in a EVOS fluorescence microscope at 100X, scale bars are shown. ***p: 0.0002.

The phagocytic index of IFN-γ activated J774 with *P. brasiliensis* was significantly increased in the presence of mAbF1.4, as showed in **Figure 2A**. In **Figure 2B**, we observed that the enhanced phagocytosis with mAbF1.4 was associated with significant increases in NO concentration as well as an enhanced fungicidal effect, determined by the killing assay and observed in **Figure 2C**. To visualize the phagocytosis assays, we performed an immunofluorescence staining. In **Figure 2D**, macrophages challenged with mAbF1.4 opsonized yeasts were labeled with a red fluorescent dye, which demonstrated the formation of phagolysosomes. The bound mAbF1.4 was marked with a green fluorescent (GFP) dye, and *P. brasiliensis* yeasts cells were visualized by blue labeling with Calcofluor white. When DAPI, GFP and RFP channels were merged, the image showed green labeled yeast cells inside of red phagolysosomes, and in some cases a yellowish fluorescence due to the superposition of the fluorescence. Yeasts labeled in blue were not phagocyted or opsonized.

### Evaluation by Fungal Burden, Pulmonary Cytokine Profile, and Lung Histopathology

**Figure 3:**
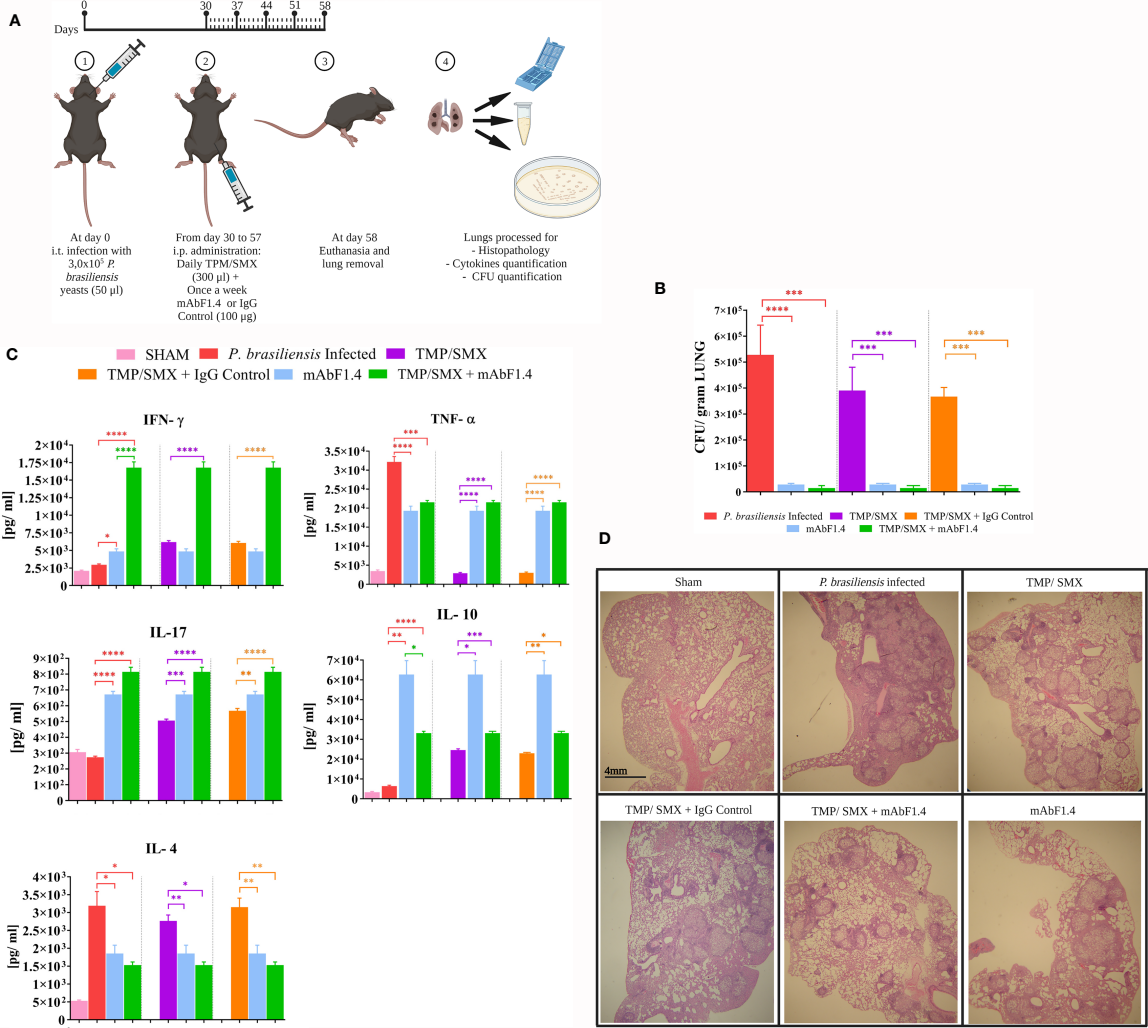
*In vivo* experiments to determine the efficacy of combined immunotherapy with mAbF1.4 and TMP/SMX. Bars depict means and SEMs of three independent experiments. Overall differences in the variance of all treatments were assessed for significance by repeated-measures one-way ANOVA, with results of Tukey’s *post hoc* test for multiple comparisons displayed in the graph, ****P < 0.0001. **(A)** Immunotherapy protocol with the TMP/SMX and mAbF1.4 represented. Groups (n = 5) were randomly organized accordingly: (i) infection control: mice infected with 3,0 ×10^5^ Pb18 yeast cells; (ii) Pb 18 infected and treated with TMP/SMX; (iii) Pb 18 infected and treated with TMP/SMX + mAb F1.4 (400 µg); (iv) infected and treated with TMP/SMX + IgG Control (mAb4G2; 400 µg); (v) Sham (uninfected, untreated). After 30 days of infection, animals were treated intraperitoneal via, daily with TMP/SMX (NeoQuímica) at 15 mg/kg/SMX: 3 mg/kg dose and once a week (100 µg) mAbF1.4, for 4 weeks. By day 58 the animals were euthanized, lungs were sterilely extracted and processed. Created with Biorender.com. **(B)** Evaluation by pulmonary fungal burden. Graphic shows the CFU/gram of tissue, determined by the viable yeasts number present in lungs macerate for every group. **(C)** Evaluation by pulmonary cytokine profile of the experimental groups. Supernatants from lung macerated were assayed for cytokines IL-4, IL-10, IL-17, TNF-α and IFN-γ by ELISA. The graphic shows a comparison of the treatments (TMP/SMX + mAbF1.4 and mAbF1.4) vs. the control groups (infected non treated, TMP/SMX and TMP/SMX + IgG control). **(D)** Evaluation by lung histopathology of sections from mice groups at 4X. Sections slides were stained with hematoxylin and eosin staining to evaluate morphology, inflammation and the granuloma formation. *p 0.01; **p:0.001 ; ***p:0.0001.

In **Figure 3A** we illustrated the therapeutic protocol performed in this study. We determined that combination immunotherapy with TMP/SMX + mAbF1.4 significantly decreased the lungs colony- forming units when compared with the control treatments: TMP/SMX and TMP/SMX + irrelevant mAb groups, as it is depicted in **Figure 3B**. It is relevant to highlight that the groups treated with TMP/SMX + mAbF1.4 and mAbF1.4 alone did not show a significant difference in the fungal load. Therefore, both treatments showed an important decrease in the CFU when compared with the control treatments.

In **Figure 3C** the pulmonary cytokine profile determined by quantitative ELISAs showed an increase in the INF- γ, TNF- α, IL-10, and IL-17 titers, as well as a decrease in IL-4 titers of the animals treated with TMP/SMX + mAbF1.4 when compared to the mice groups treated with TMP/SMX alone and TMP/SMX + irrelevant mAb. These results suggest a Th1 and Th17 immune responses modulated by the immunotherapy with TMP/SMX in combination with mAbF1.4. The mice treated with mAbF1.4 alone showed INF- γ and TNF- α titers similar to the TMP/SMX, or TMP/SMX + irrelevant mAb treated mice, as well as IL-4 titers similar with TMP/SMX + mAbF1.4 treated mice. Interestingly, the mice treated with mAbF1.4 alone exhibited IL-10 titers significantly increased compared with all treatments, while IL- 17 titers were higher when compared to TMP/SMX, or TMP/SMX + irrelevant mAb treated mice, but lower than TMP/SMX + mAbF1.4 treated group. Curiously, the mice treated with TMP/SMX and TMP/SMX with irrelevant mAb had TNF-levels resemblant with sham mice. However, TMP/SMX and TMP/SMX with irrelevant mAb treatments increased the INF- γ, IL-10, and IL-17 titers when compared to the *P. brasiliensis* infected- untreated group, while IL-4 titers showed no significative difference with the same group.

In **Figure 3D** HE lung tissue analysis shows histopathology of lung sections of all treatment groups studied. The mice infected with *P. brasiliensis* exhibited large inflammation and diffuse granulomas. However, we confirmed that combination treatment of TMP/SMX + mAbF1. resulted in the preservation of the pulmonary architecture, in a decrease of the granulomatous inflammatory response with less pulmonary fibrosis, and in yeast cells confined within granulomas when compared to the lung sections of mice infected with *P. brasiliensis* untreated, and with the mice that received TMP/SMX or TMP/SMX + irrelevant mAb. Notably, mice treated with TMP/SMX alone had decreased inflammation and compact granulomas when compared to the *P. brasiliensis* infected- untreated group. The mice treated with mAbF1.4 alone displayed larger granulomas and more inflammation when compared with the TMP/SMX + mAbF1.4 group. However, this group exhibited lower inflammatory response with larger granulomas when compared with the TMP/SMX alone treated mice.

## Discussion

The fungal cell wall is a key point of early interaction with the host during infection and colonization, since it is the most external structure (28), with the exception of certain encapsulated organisms, like *Cryptococcus* sp. Fungal cell walls are typically composed by β-glucans (around 50-60%), glycoproteins (around 20-30%) and a minor percentage of chitin (17). In the case of *P. brasiliensis*, the maintenance of the cell wall is particular (29). The content of α- and β-glucans are genetically modulated according to temperature and atmospheric conditions, since *Paracoccidioides* spp. are thermal dimorphic fungi. (8, 30). When *P. brasiliensis* switch the morphology from the mold (18–25°C) to the yeast form (35–37°C) the proportions of α- and β-glucans also changes. The cell wall of *Paracoccidioides* spp. yeast cells is composed of diverse macromolecules containing polysaccharides, among them α- and β-glucans covalently linked to proteins, amino acids or lipids, which are known as glycoconjugates (31–33). The mycelial form of *Paracoccidioides* spp. is differentiated for displaying mostly β-1,3-glucan polymers on the cell wall. Even though the cell wall of the yeast form is characterized for showing greater proportions of α-1, 3-glucan (95% of total glucan), than β-glucans (~5%) (34), it is known that α- glucans have limited immunogenicity on mammal hosts (35). The β-glucan network is highly complex, including the covalent binding of β1,3-glucans to β1,6-glucans and to chitin (19). Many cell wall proteins are covalently linked to a glycosylphosphatidylinositol (GPI) anchor and these GPI-anchored proteins are covalently linked to cell wall β1,6-glucan through a truncated GPI remnant (31, 32, 36). Remarkably, polysaccharides can moderate the pathogenesis of systemic mycosis *via* numerous mechanisms (37). Torosantucci et al., demonstrated that β- glucans are capable of eliciting protective antibody responses against fungal infections in a model using a prophylactic approach (38).

We performed a dot blot assay to characterize the polysaccharidic binding residues recognized by mAbF1.4. Using sequence-defined glucan oligosaccharides, we discovered that mAbF1.4 selectively and efficiently bound to both, β-1,3/β-1,6-linked glucose residues present in a laminarin polymer and the *S. cerevisiae* β-glucan (27). In contrast, there was significantly lower binding with the linear polymer of β-1,3-linked glucose residues (zymosan). It is important to consider the cell wall architecture and localization of the β-glucan epitopes. GPI-Anchored proteins are commonly linked to the cell wall by a β-1,6/β-1,3- glucan (19, 39). Our hypothesis is that mAbF1.4 recognizes β-1,3/β-1,6-linkage glucose residues that are typically found on the cell wall of fungi and may anchor GPI-proteins to the cell wall. Future experiments will further elucidate the specific fine details of the epitope for mAbF1.4. Also, we will determine if mAbF1.4 binds to other fungi species of medical importance

Obtaining natural purified β-glucan antigens to produce and select protective mAbs is a complex challenge, since the fungal cell wall is highly dynamic and constantly alters its polysaccharide composition and disposition, showing divergent immunogenic properties (40). We generated mAbF1.4 using glycoconjugate extracts from *in vitro* cultivated yeast cells. For *in vitro* evaluation, we analyzed if mAbF1.4 could bind to live *P. brasiliensis* yeasts cells and modulate their phagocytosis by macrophages, since macrophages are among the first line of host defense against *Paracoccidioides* spp. infection (41, 42). After the identification of fungal cell wall polysaccharides, fast immune responses such as phagocytosis, production of antimicrobial compounds, and induction of proinflammatory cytokines by macrophages are induced, leading to the activation of other immune effector cells (43, 44). We determined that mAbF1.4 modulated the enhanced phagocytosis of *P. brasiliensis* yeasts, increasing the phagocytic index. Nitrosative stress is characterized by increased oxygen absorption and increased production of both reactive oxygen species (ROS) and reactive nitrogen species (RNS), which are involved in the killing of phagocytosed yeasts (41, 43, 45). The nitrite concentration from the phagocytosis supernatant samples increased in macrophages challenged by mAbF1.4 opsonized yeast cells, when compared to macrophages challenged with non-opsonized or irrelevant mAb opsonized *P. brasiliensis* yeasts. Also, the increased concentration of nitrite was associated with a significant reduction in the CFU from the macrophages co-cultured with yeast opsonized with mAbF1.4. Our results show that mAbF1.4 enhanced the phagocytosis of opsonized yeasts and increased NO production and excretion by activated macrophages, inducing a nitrosative stress that produced an efficient fungicidal effect.

Currently, all the available antifungal agents are toxic to humans, because of the similarities between mammal cells and fungi. Regarding PCM treatment, extended periods of antifungal therapies, infection relapses, and constant medical monitoring highlight the urgency for the development of new therapeutic alternatives, mainly strategies based on the enhancing of the immune responses (46, 47). Moreover, the research for therapeutic approaches that aim to improve the immune system of hosts suffering of impaired immune responses is a challenge that must be pursued in order to enhance the effectiveness of the available antifungal therapies (4, 48).

In order to test the efficacy of an alternative therapy to PCM, we combined TMP/SMX, the most frequently antimycotic administered in Brazil to treat PCM, with mAbF1.4 to treat experimental chronic PCM. *In vivo*, we determined that immunotherapy with TMP/SMX in combination with mAbF1.4 efficiently reduced the *P. brasiliensis* pulmonary fungal burden of C57Bl/6 mice with PCM, since this therapy significantly decreased the colony- forming units. Interestingly, there was no statistically significant difference in CFU between the mice treated with mAb alone or both mAb and TMP/SMX, although there was a trend toward the combination being more potent. In addition to facilitating opsonophagocytosis, mAbs have several other potentially impactful effects that may be pertinent to mAbF1.4’s efficacy. Binding to components of the cell wall by antibody can inhibit yeast cell growth (49), affect membrane remodeling (50), change gene regulation (51, 52), and alter the secretion of virulence factors, including those packaged into and released in extracellular vesicles (53, 54). However, the impact of the combination is highlighted by shifts in protective specific cytokines and beneficial tissue effects.

In murine PCM models, granulomas are well developed at four-week post-infection, when the fibers of collagen and reticulin become visible and the presence of leukocyte infiltrates surrounding the granuloma is evident (55, 56). At least 60% of patients suffering chronic PCM develop pulmonary fibrosis and, consequently, respiratory function loss (8, 9). The use of antifungal drugs for long periods of time in the treatment of chronic PCM does not affect the progression of pulmonary fibrosis, which remains even after the antifungal therapy is completed (56). We found on the histopathology slides of lung segments that the mice group treated with TMP/SMX in combination with mAbF1.4 showed reduced number and well-defined lung granulomas, and more preserved lung architecture in comparison with the other infected groups. Notably, mice that were treated with TMP/SMX alone had increased granuloma formation than the mice group treated with TMP/SMX in combination with mAbF1.4 or mAbF1.4 alone. Previous publications by our research group demonstrated PCM protection *in vivo* associated with the induction of a Th1-type immune response, especially characterized by increased production of cytokines IL-2, IL-12 and IFN-γ and decreases in IL-4 and IL-10 (26, 46, 57). In this work, we observed a mixed Th1- th-17 type immune response in mice treated with SMX/TMP and mAbF1.4.

The nature of the mechanisms that trigger the modulation of PCM in mice treated with mAbF1.4 is a complex topic. However, we propose the hypothesis that mAbF1.4 binds to glycoconjugates present in the cell wall β-glucans and the opsonization of the yeast may then boost the phagocytosis and killing of the *Paracoccidioides* cells by macrophages that have increased production of IFN-γ and TNF-α production. The data suggests that mAbF1.4 is a potent immunomodulator due to its ability to interact with effector cells to shift the inflammatory response. In future work, we will explore the efficacy of mAbF1.4 in different strains and species of *Paracoccidioides* as well as to test binding of the mAb to other pathogenic fungi, since β-1,3/β-1,6-linked glucose residues are present in other important causes of human mycoses (37). This work demonstrates the promise of synergistic therapy of a standard antifungal with a glycoconjugate-binding mAb for combating chronic PCM.

## Data Availability Statement

The original contributions presented in the study are included in the article/**Supplementary Material**. Further inquiries can be directed to the corresponding authors.

## Ethics Statement

The animal study was reviewed and approved by Animal Use Ethics Committee (CEUA- ICB protocol number 66/2017), Instituto de Ciências Biomédicas, Departamento de Microbiologia, Universidade de São Paulo, São Paulo, Brazil.

## Author Contributions

CB: Conceptualization, methodology, software, validation, formal analysis, investigation, data curation, project administration, and writing—original draft preparation. BK: methodology, investigation, and validation. MG: Methodology, investigation, and validation. LT: Methodology, investigation, and validation. JN: Writing and editing. LL-B: Formal analysis, writing, and editing. CT: Supervision, funding acquisition, and manuscript editing. All authors have read and agreed to the published version of the manuscript.

## Funding

This research was supported by FUNDAÇÃO DE AMPARO A PESQUISA DO ESTADO DE SÃO PAULO (FAPESP), grants number 2016/08730-6, 2018/26402-1 and Conselho Nacional de Desenvolvimento Científico e Tecnológico (CNPq) grant number 420480/2018-8.

## Conflict of Interest

The authors declare that the research was conducted in the absence of any commercial or financial relationships that could be construed as a potential conflict of interest.

## Publisher’s Note

All claims expressed in this article are solely those of the authors and do not necessarily represent those of their affiliated organizations, or those of the publisher, the editors and the reviewers. Any product that may be evaluated in this article, or claim that may be made by its manufacturer, is not guaranteed or endorsed by the publisher.
