# Supplementary material for: Antibody- Based Immunotherapy Combined With Antimycotic Drug TMP- SMX to Treat Infection With Paracoccidioides brasiliensis
Source: Front Immunol. 2021 Oct 19;12:725882. doi: 10.3389/fimmu.2021.725882 (PMC8562153; doi:10.3389/fimmu.2021.725882)
Supplement: Supplementary file 1 [file DataSheet_1.pdf]

Supplementary Material

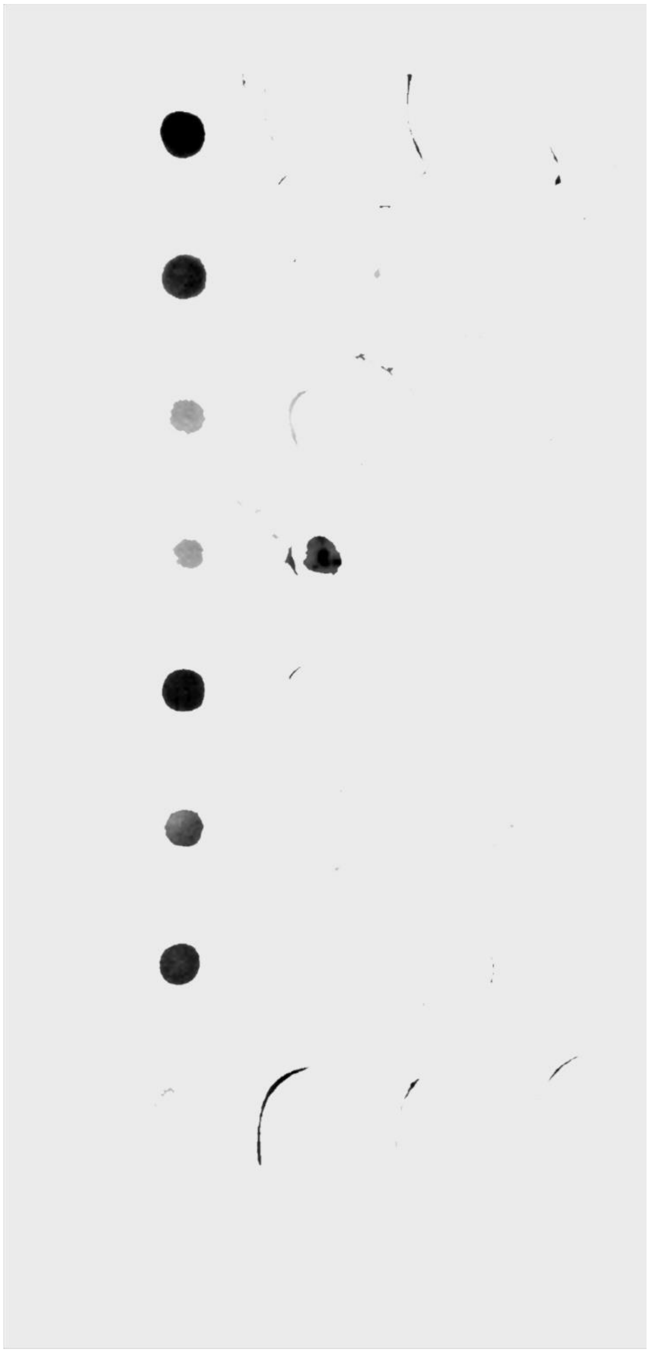

Dot blotting performed from which the images in **Figure 1 (A)** were cropped. In the first line, 2 samples (100 µL) of laminarin (0.1 µg/µL), 2 samples of zymosan (0.1 µg/µL), 3 samples of soluble β-glucan extract from *Saccharomyces cerevisiae*, and 2 samples of yeast mannan (0.1 µg/µL) were tested with mAbF1.4 (10 µg). In the second line, the same samples were tested with the irrelevant mAb (10

μg) as negative control. An anti-mouse IgG Horseradish Peroxidase at 1:2500 dilution was visualized with Peroxide and immediately imaged in a documentation system. The dark spots in Laminarin, Zymosan and soluble β-glucan extract reactions with mAbF1.4 evidence antibody- antigen complexes. The spot in the second line where the negative controls were tested correspond with an extra sample of soluble β-glucan extract from *Saccharomyces cerevisiae* tested with mAbF1.4.

2 Supplementary Figures and Tables

Supplementary Table 1. Monosaccharide profile of the glycoconjugate cell wall fraction determined by gas chromatography.

**Monosaccharide proportion**	**Arabinose**	**Xylose**	**Mannose**	**Galactose**	**Glucose**
P. brasiliensis (Pb18)	0	0	11%	7%	79%

Samples of glycoconjugate cell wall fraction extracted from *P. brasiliensis* yeasts were hydrolyzed and acetylated, their composition was determined as alditols.

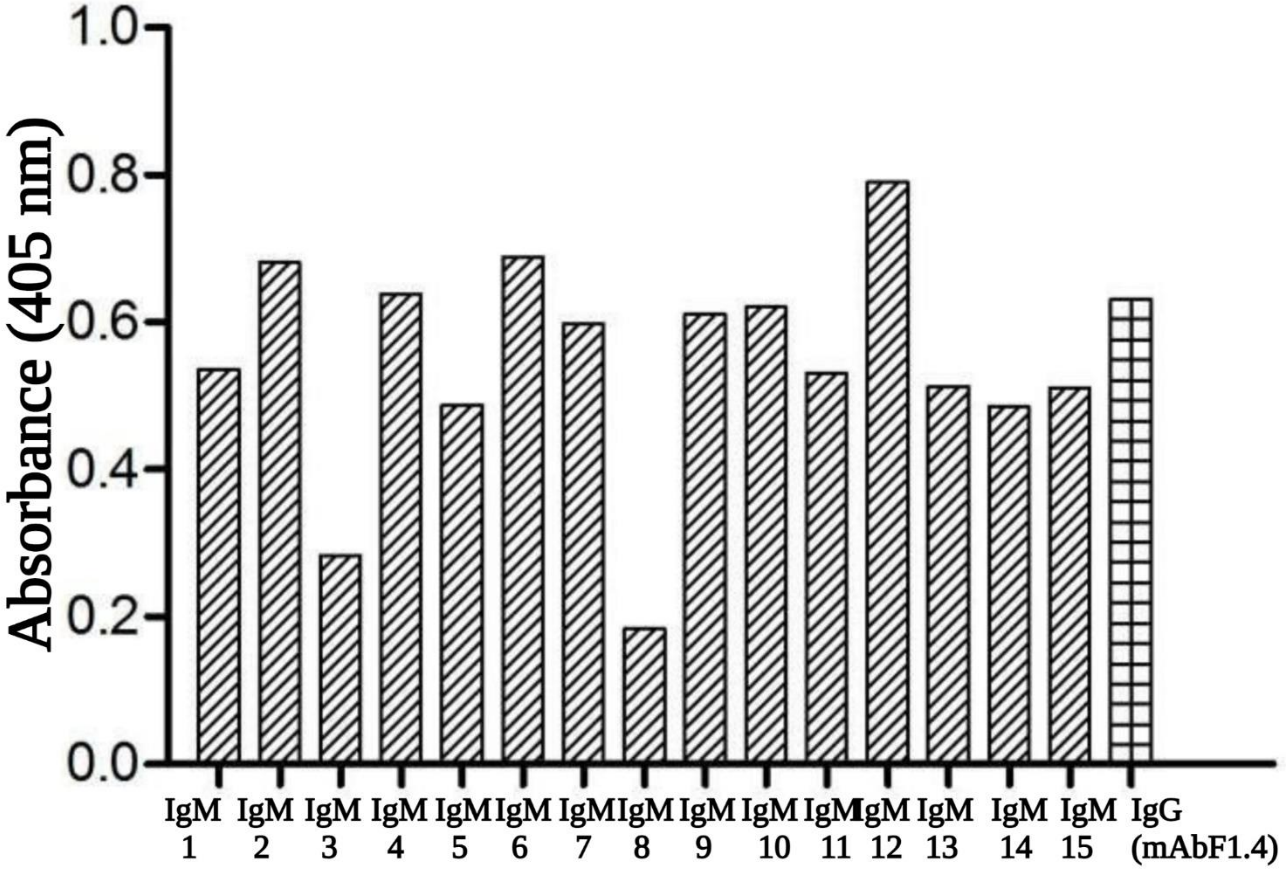

Supplementary Figure 2. Reactivity of mAbF1.4 against the glycoconjugate cell wall fraction determined by ELISA at 1:100 dilution. The graphic shows all the clones obtained by hybridoma technology against the glycoconjugate cell wall fraction extracted from *P. brasiliensis* yeasts.
